# Primary Intrathoracic Synovial Sarcoma: An Analysis of Outcomes of This Rare Disease

**DOI:** 10.3390/cancers17050745

**Published:** 2025-02-22

**Authors:** Riddhi R. Patel, Andrew J. Bishop, Alexander J. Lazar, Patrick P. Lin, Robert S. Benjamin, Shreyaskumar R. Patel, Joseph Ludwig, Vinod Ravi, Ara A. Vaporciyan, Dejka M. Araujo

**Affiliations:** 1 Department of Sarcoma Medical Oncology, The University of Texas MD Anderson Cancer Center, 1515 Holcombe Blvd. Unit 450, Houston, TX 77030, USA; rpatel9@mdanderson.org (R.R.P.); rbenjami@mdanderson.org (R.S.B.); spatel@mdanderson.org (S.R.P.); jaludwig@mdanderson.org (J.L.); vravi@mdanderson.org (V.R.); 2 Department of Epidemiology, The University of Texas Health Science Center, Houston, TX 77030, USA; 3 Department of Radiation Oncology, The University of Texas MD Anderson Cancer Center, Houston, TX 77030, USA; abishop2@mdanderson.org; 4 Department of Pathology, The University of Texas MD Anderson Cancer Center, Houston, TX 77030, USA; alazar@mdanderson.org; 5 Department of Genomic Medicine, The University of Texas MD Anderson Cancer Center, Houston, TX 77030, USA; 6 Department of Orthopedic Oncology, The University of Texas MD Anderson Cancer Center, Houston, TX 77030, USA; plin@mdanderson.org; 7 Department of Thoracic and Cardiovascular Surgery, The University of Texas MD Anderson Cancer Center, Houston, TX 77030, USA; avaporci@mdanderson.org

**Keywords:** synovial sarcoma, intrathoracic, localized disease

## Abstract

**Background:** Primary intrathoracic synovial sarcoma (SS) is a rare entity. The objective of this study was to evaluate survival outcomes for patients with intrathoracic SS presenting with localized disease at diagnosis. **Methods:** We conducted a retrospective review of 63 patients diagnosed with intrathoracic SS between 1997 and 2020. The Kaplan–Meier method and log-rank test were used to estimate the progression-free survival (PFS), overall survival (OS), local recurrence-free survival (LRFS), and metastasis-free survival (MFS). The hazard ratios were estimated by using Cox proportional hazards regression. Median follow-up time, age-at-diagnosis, and primary tumor size were 31 months (range: 4–218 months), 43 years (range: 18–77), and 7 cm (range: 1–23), respectively. **Results:** Sixty-two of sixty-three (98%) patients had their primary tumor resected, from whom eighteen (29%) and forty-three (69%) had received neo/adjuvant radiotherapy and chemotherapy, respectively. Median PFS, OS, and MFS were 1.2, 3.0, and 1.1 years, respectively. Based on multivariable analyses, patients with ≥5 cm tumor size had poorer OS (versus < 5 cm; HR: 2.66; 95% CI: 1.16, 6.11; LR-*p* = 0.014). Importantly, the receipt of neo/adjuvant chemotherapy was the only factor associated with both a more favorable PFS (HR: 0.33; 95% CI: 0.17, 0.65; LR-*p* = 0.0002) and a more favorable MFS (median 1.33 years versus no chemo 0.5 years; HR: 0.35; 95% CI: 0.17, 0.73; LR-*p* = 0.005). **Conclusions:** Outcomes associated with intrathoracic SS remain poor. Factors associated with poorer outcomes include larger tumors and omission of chemotherapy in the management of localized disease. We recommend providing perioperative chemotherapy to all patients with ≥5 cm tumor size to improve progression and metastasis-free survival.

## Simple Summary

Intrathoracic synovial sarcoma is a rare type of cancer, and little is known about the survival outcomes of patients diagnosed with localized disease. This study aims to assess multiple survival outcomes and their predictors using the largest group of patients with localized intrathoracic synovial sarcoma. By reviewing data from 63 patients, we found that larger tumors were linked to worse overall survival, while chemotherapy before or after surgery improved both progression-free and metastasis-free survival. These findings suggest that chemotherapy should be considered for all patients with tumors 5 cm or larger to improve their chances of better outcomes. Understanding these factors may help doctors make more informed treatment decisions for patients with this rare cancer.

## 1. Introduction

Synovial sarcoma (SS) is a rare cancer accounting for about 10% of all soft tissue sarcomas [1]. SS is classified histologically as monophasic (spindle cells only), biphasic (spindle cells and epithelial cells), or poorly differentiated. In addition to histologic characteristics, the pathognomonic SS18::SSX1/2 fusion genes are a critical diagnostic marker for SS. It usually affects middle-aged adults with a slight male predominance and occurs commonly in the extremities [2].

Although pulmonary metastases are common in patients with soft tissue sarcomas, primary sarcomas of the chest are rare, and primary sarcomas of the lung are even rarer. The most common sarcomas arising in the chest involve the heart (angiosarcomas on the right, undifferentiated pleomorphic sarcomas on the left), great vessels (intimal sarcomas and venous leiomyosarcomas), and mediastinum (well-differentiated and dedifferentiated liposarcomas). SS may be the most common sarcoma that arises in the lung. Although the chest is a relatively rare primary site for SS, primary SS of the lung and mediastinum have been reported, although a series of more than a few cases is unusual [3,4,5,6]. When they do arise in the intrathoracic cavity, origins in the lung and pleura are the most common locations, followed by the mediastinum and heart [3,7]. Patients with intrathoracic SS often present with advanced disease and symptoms that include difficulty breathing, chest pain, cough, and pleural effusion [4,8,9].

Given the rarity of the diagnosis, the limited literature on these tumors is comprised predominantly of case reports or small case series mainly characterizing the clinicopathological and immunohistochemical features [5,6,7,10,11,12,13,14]. Clinical outcomes have been described in only a few series with a limited number of cases [3,4,15,16,17]. Past studies have explored the association between tumor location and survival among SS patients in general [18,19], but tumor origin within the chest has not been assessed yet. Additionally, as the median survival time for SS patients with metastasis at diagnosis is only 1.3 years [20], it is imperative to identify factors associated with better outcomes. As the behavior of SS is different with different primary sites and given our synovial sarcoma program, we sought to examine the largest cohort published to date of patients with intrathoracic SS presenting with localized disease to better describe outcomes and to evaluate any associations between tumor origin within the chest and patient outcomes.

## 2. Materials and Methods

A retrospective chart review was conducted after the approval by the University of Texas MD Anderson Cancer Center (MD Anderson) Institutional Review Board. Patients diagnosed with intrathoracic SS between 1997 and 2020 who had localized disease at the time of diagnosis and who received treatment for primary or metastatic cancer at this comprehensive cancer center were included in the study. Data on patients’ demographic and clinical characteristics were obtained through electronic medical records. Histologic confirmation of the SS diagnosis was conducted by soft tissue sarcoma pathologists.

### Statistical Method

The following time-to-event outcomes were analyzed: overall survival (OS), defined as the time interval between diagnosis and death from all causes; progression-free survival (PFS), defined as the time interval between diagnosis and local recurrence/metastasis; local recurrence-free survival (LRFS), defined as the time interval between completion of the definitive treatment for primary tumor and first local recurrence; and metastasis-free survival (MFS), defined as the time interval between completion of the definitive treatment for primary tumor and first metastasis. The surgical resection+/− radiation therapy (RT) was considered as a definitive treatment of the primary tumor. In order to define both LRFS and MFS, the time of origin for patients who received postoperative radiation was the end date of RT, and for those who received preoperative or no radiation, it was the date of surgery. Various patient and tumor-related characteristics were categorized, including tumor origin/extent (lung, lung with involvement of pleura, chest wall, and mediastinum), age at diagnosis (<45, ≥45 years), sex (male, female), race and ethnicity (White, other), tumor size (<5, ≥5 cm), stage at diagnosis (stage ll, lllA, lllB), surgical margin after final primary tumor surgery (positive, negative, unknown), surgery for primary tumor (yes, no), RT received with primary tumor surgery (yes, no), and chemotherapy received with primary tumor surgery (yes, no). SS cases with outer chest wall tumor location were excluded from the study. Other race and ethnicity categories included Hispanic or Latino and Asian. Stage at diagnosis was classified based on the *American Joint Committee on Cancer (AJCC) 8th Edition* [21]. Three variables, surgical margin, neo/adjuvant RT, and neo/adjuvant chemotherapy, were calculated only for those patients who had undergone primary tumor resection. If any variable had missing data on more than 15% of the population, an “Unknown” category was created, such as for surgical margin, to prevent reducing the sample size and power of the study.

The Kaplan–Meier method was used to analyze time-to-event data (OS, PFS, LRFS, and MFS), and the log-rank test was used to test the difference in survival curves between groups. Patients who were lost to follow-up were censored at the last date of contact for all the time-to-event calculations. The univariate and multivariable Cox proportional hazards regression models were used to evaluate the independent effects of the predictors on survival. The multivariable models included significant variables from univariate analysis. There were two multivariable analyses: (1) to evaluate the effect of tumor origin on OS and (2) to evaluate the effect of tumor origin on PFS. All the analyses were conducted using Stata 17.0 (Stata-Corp, College Station, TX, USA); *p* < 0.05 was considered for statistical significance.

## 3. Results

We identified 63 patients with intrathoracic SS who presented with localized disease at the time of diagnosis. The median follow-up time was 31 months (range: 4–218 months). Table 1 presents the demographic and clinical characteristics of the intrathoracic SS patients. The median age at diagnosis was 43 years (range: 18–77). The proportion of female patients was a little higher (57%) than males (43%), and most patients were White (n = 57, 90%). In addition, 30% of the patients had a history of smoking.

Lung was the most common intrathoracic primary tumor site (n = 43, 68%), followed by the mediastinum (n = 15, 24%) (Table 1). The median primary tumor size was 7 cm (range: 1–23), and 40 of 63 (63%) patients had a tumor size larger than 5 cm (Table 1). The proportion of patients with monophasic SS (n = 47, 75%) was higher as compared to those with biphasic or poorly differentiated SS (Table 1). A majority of patients who underwent surgical resection had negative resection margins (56%) (Table 1). Additionally, 62 of 63 (98%) patients had their primary tumor resected, of whom 29% (n = 18) had received neo/adjuvant RT and 69% (n = 43) chemotherapy. The only patient who did not undergo resection had a 4.9 cm poorly differentiated SS of the mediastinum with associated mass effect on the posterior heart and vasculature. The patient was treated with chemotherapy followed by RT.

The most common first-line chemotherapy regimen was doxorubicin and ifosfamide for both neoadjuvant (n = 13 out of 16, 81%) and adjuvant (n = 21 out of 26, 81%) therapy. The most common side effects of this regimen included neutropenic fever and prolonged thrombocytopenia, followed by anemia, severe pancytopenia, colitis, and torsades with cardiac arrest. The second and third most common regimens included high-dose ifosfamide, single-agent doxorubicin, a combination of doxorubicin and dacarbazine, or a combination of gemcitabine and docetaxel. The median number of neoadjuvant chemotherapy cycles was 4.5 (range: 1–6), and of adjuvant chemotherapy cycles was 6 (range: 2–9) in the patients who underwent primary tumor resection.

**Figure 1:**
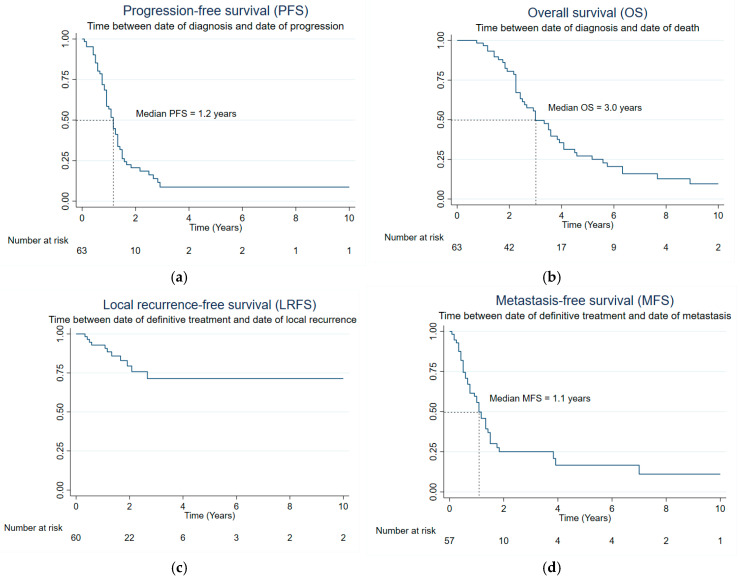
Progression-free survival (**a**) and overall survival (**b**) curves for the entire cohort (n = 63); and local recurrence-free survival (**c**) and metastasis-free survival (**d**) curves for intrathoracic SS patients presenting with localized disease who had surgical resection (n = 62).

Of the 63 patients, 45 (71%) have died. Median PFS and OS were 1.2 and 3.0 years, respectively (Figure 1a,b). The estimated 1, 3, and 5-year PFS rates were 57% (95% CI: 43%, 68%), 8% (95% CI: 3%, 19%), and 8% (95% CI: 3%, 19%), respectively. The estimated 1, 3, and 5-year OS rates were 97% (95% CI: 88%, 99%), 50% (95% CI: 36%, 62%), and 27% (95% CI: 16%, 40%), respectively.

In addition, the median LRFS was not reached, whereas the median MFS was 1.1 years (Figure 1c,d). The estimated 1, 3, and 5-year LRFS rates were 93% (95% CI: 82%, 97%), 71% (95% CI: 53%, 84%), and 71% (95% CI: 53%, 84%), respectively. The estimated 1, 3, and 5-year MFS rates were 56% (95% CI: 41%, 68%), 25% (95% CI: 14%, 38%), and 17% (95% CI: 7%, 31%), respectively.

**Figure 2:**
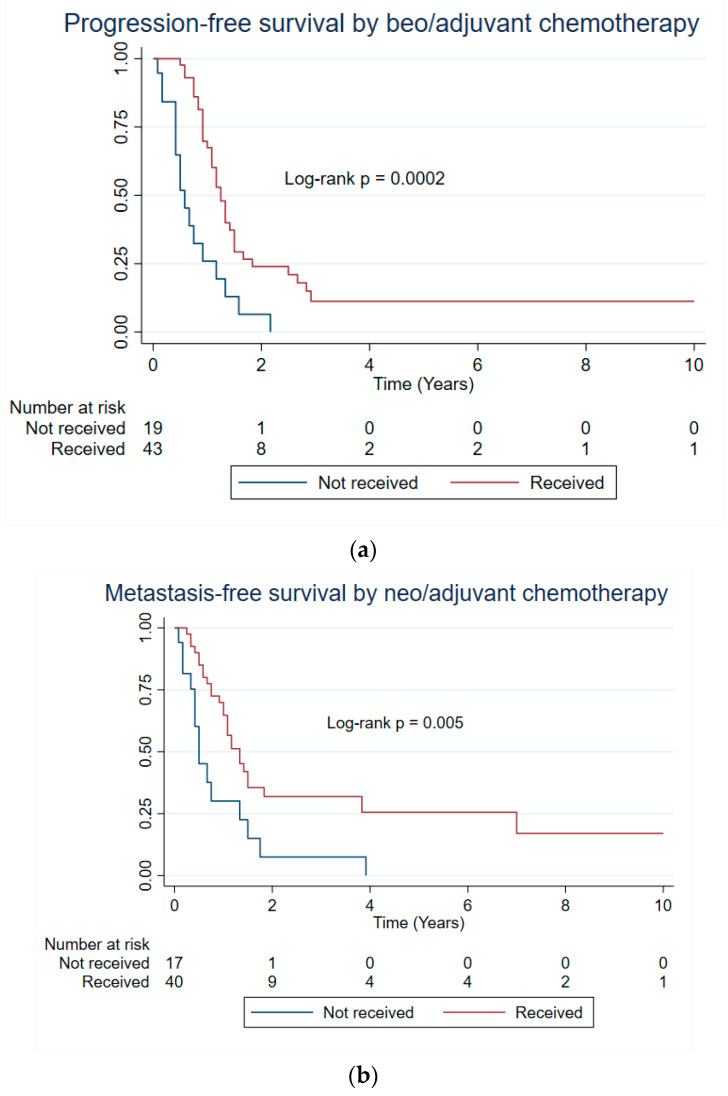
Progression-free survival (**a**) and metastasis-free survival (**b**) by neo/adjuvant chemotherapy for intrathoracic SS patients presenting with localized disease (n = 63).

**Figure 3:**
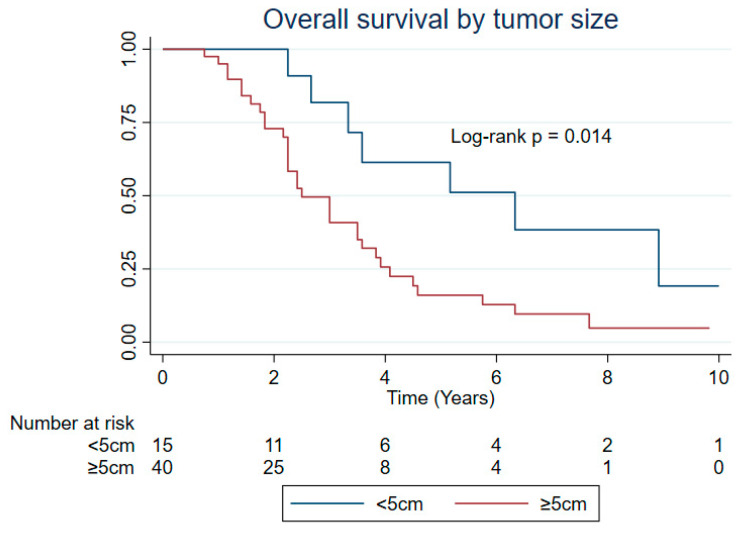
Overall survival by primary tumor size for intrathoracic SS patients presenting with localized disease (n = 63).

Table 2 presents the median survival time with log-rank *p* values, and Table 3 presents the hazard ratios (HRs) and 95% Confidence Interval (CI) for all the survival outcomes. Based on the multivariable analysis, patients who received neo/adjuvant chemotherapy had 67% less hazard of progression than those who did not receive it (HR: 0.33; 95% CI: 0.17, 0.65; LR *p* = 0.0002) (Table 2 and Table 3, and Figure 2). Moreover, patients with ≥5 cm tumor size had 2.66 times the hazard of dying (95% CI: 1.16, 6.11; LR *p* = 0.014) than those with less than 5 cm tumor size (Table 2 and Table 3, and Figure 3). As there was no significant difference in OS for patients with 5–10 and ≥10 cm tumors (Supplementary Figure S1, *p* = 0.73), tumor size was categorized into <5 and ≥5 cm for the analysis.

To evaluate the effect of neo/adjuvant RT on local control (LRFS) and the effect of neo/adjuvant chemotherapy on MFS, multivariable Cox proportional hazard regressions were conducted for patients with surgical resection (n = 62). There was no significant predictor found for LRFS. However, patients who received chemotherapy with surgical resection had a 65% lower hazard of metastasis after definitive treatment for the primary tumor than those who did not receive it (HR: 0.35; 95% CI: 0.17, 0.73). (Table 3). Also, the median MFS for patients who received neo/adjuvant chemotherapy was better (1.33 years) than for those patients who did not receive it (0.50 years; LR *p* = 0.005) (Table 2 and Figure 2).

## 4. Discussion

Here, we report on the largest and most homogenous cohort of patients with SS of the intrathoracic cavity. Overall, our study revealed poor outcomes despite a localized presentation. We observed no association between the tumor origin within the chest and survival. Importantly, the use of neo/adjuvant chemotherapy was associated with more favorable PFS and MFS, whereas larger tumor size was a predictor of poorer OS among patients presenting with localized intrathoracic SS at the time of diagnosis.

The estimated 5-year OS rate of 27% for intrathoracic SS patients presenting with localized disease in the current study is similar to that reported by He et al. in their recent publication [17]. Such a lower survival rate may be attributed to the unique anatomy of the lungs, the potential for easy spread within the lungs, and perhaps something pertaining to the primary tumor origin already primed to grow in the lungs. Importantly, a 5-year OS of 27% in intrathoracic SS patients was remarkably lower than that of other sites such as the head and neck (70%) [22], and hand and foot (88%) [23]. This may be due to relatively higher chances of delayed tumor detection in the chest than in the extremities. There are also certain challenges associated with the treatment of a primary intrathoracic SS because of the anatomic location and complexity of the surgical resections.

The median OS and PFS in our cohort were 3.0 years and 1.2 years, respectively. Similarly, He et al. also noted a median OS of 46 months (3.8 years) among patients managed by a multidisciplinary team, which is the same approach of treatment at our institution [17]. Likewise, Begueret et al. also found the median disease-specific survival to be 50 months (4.2 years) [16]. The same study also observed a median disease-free survival of 24 months [16], that was a little higher than 1.2 years (14 months) of median PFS in our study. Notably, the 2-year LRFS was 79% in our study; in other words, the 2-year cumulative local recurrence rate was 21%, which was close to that reported by Zeren et al. (22%) [24], Begueret et al. (27.2%) [16], and Okamoto et al. (30%) [5].

We also evaluated the distributions of various demographic, tumor, and treatment characteristics as part of the disease epidemiology. Even though SS is generally more common in males, this study had relatively more female intrathoracic SS patients. However, sex was not a significant predictor of survival in our study. Previous studies performed by Paul Hartel et al. and Gun Ha Kim et al. also did not show significant sex predominance related to intrathoracic SS [4,14].

Additionally, the majority of the patients in our study were White, as observed in SS from other anatomic sites [22,23]. This result is supported by the fact that SS diagnosis is more common among Whites as compared to Blacks [25]. Also, limited tertiary health care access among non-White patients may also play a role in the observed proportional difference [26]. Moreover, certain referral patterns to a tertiary cancer center may have contributed to the proportionately higher frequency of White patients.

In addition, we found a relatively higher proportion of poorly differentiated cases (8%) as compared to other tumor sites such as the head and neck (6%) [22] and other studies focusing on localized SS of multiple sites (4%) [27,28]. Concordant to our result, studies performed by Bégueret et al. and Ting et al. also had a larger proportion of poorly differentiated intrathoracic SS cases (37.5% and 11.5%, respectively) [3,16].

The primary tumor size was a significant predictor of the OS in our study. A study showing the importance of tumor size on survival for extremity and truncal SS indicated similar results that patients with tumors greater than 5 cm had an increased risk of death compared to those with tumors less than 5 cm [18]. However, a study by He et al. pertaining to only intrathoracic SS did not find any association between tumor size and survival [17]. The distinguishing factor between the two studies was that in the He report, they had a cutoff of 7 cm tumor size rather than 5 cm, which makes it hard to compare with our findings. As seen in Supplementary Figure S1 of our study, there is not much difference in survival for those with 5–10 vs. ≥10 cm tumor size, so it is reasonable to evaluate the 5 cm cutoff.

Furthermore, RT was not associated with PFS, OS, and MFS on univariate analyses (Table 3). However, neo/adjuvant RT was more commonly recommended in patients with larger tumors (*p* = 0.048) (Supplementary Table S1).

**Figure 4:**
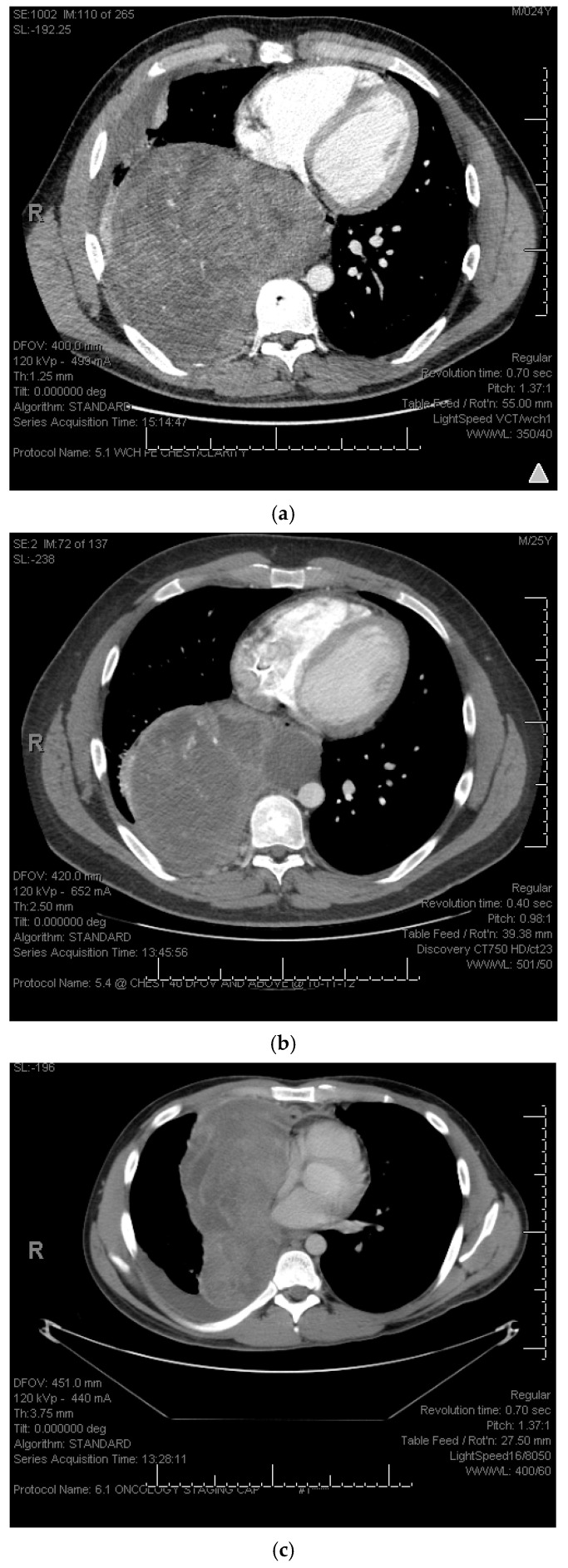

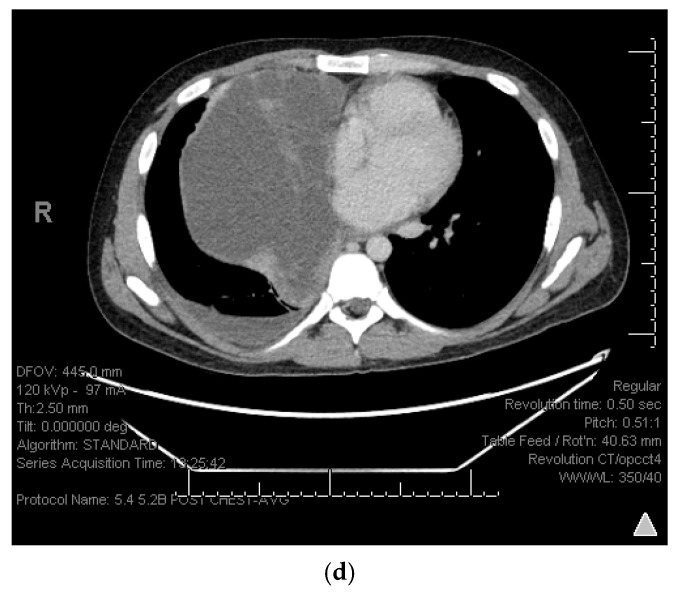
CT scans before and after chemotherapy in patients with primary lung synovial sarcoma. (**a**) Baseline CT scan. (**b**) CT scan after neo-adjuvant chemotherapy showing decreased size and increased necrosis. (**c**) Baseline CT scan. (**d**) CT scan after neo-adjuvant chemotherapy showing increased necrosis of the tumor.

Although SS is chemo-sensitive and perioperative chemotherapy is widely used in SS patients, its effect on survival has not been established yet [29,30]. The patients in our cohort who had received systemic therapy before or after surgical resection showed better prognoses related to PFS. Also, such patients had significantly higher metastasis-free time (14 months) after definitive treatment of the primary tumor. Whereas metastasis occurred in just 6 months of surgery+/−RT if they did not receive chemotherapy. A systematic meta-analysis of adjuvant chemotherapy in localized soft tissue sarcoma reported a benefit for chemotherapy in terms of metastasis-free survival (odds ratio–OR: 0.67; 95% CI 0.56–0.82; *p* = 0.0001) [31]. They showed benefits with both doxorubicin-based chemotherapy (OR: 0.69; 95% CI, 0.56–0.86; *p* = 0.001) as well as doxorubicin combined with ifosfamide (OR: 0.61; 95% CI, 0.41–0.92; *p* = 0.02) [31]. As there is not much information available on the benefits of chemotherapy in the literature specific to localized intrathoracic SS patients, our findings of favorable outcomes with perioperative chemotherapy are certainly an addition to the current literature. Figure 4 presents the CT scans before and after the chemotherapy in primary lung SS patients. Moreover, local recurrence was not the problem in our cohort, as only 14 of 64 (22%) who underwent surgical resection had local recurrence, whereas 49 of 65 (75%) had metastasis (Table 1). Metastatic sites varied from the same or opposite lungs, lung and pleura, mediastinum, bones, pancreas, liver, retroperitoneal, lymph nodes, and scalp. Considering the fact that intrathoracic SS patients die of metastatic disease, there is an imperative need not only for the initiation of neoadjuvant therapy but also for improvement in the systemic therapy that is used.

Additionally, upon assessing the impact of perioperative chemotherapy among patients with 5 cm or greater tumor size, we found that patients who received neo/adjuvant chemotherapy had 72% (HR: 0.28; 95% CI:0.13, 0.62) lower hazard of progression after diagnosis, and 73% (HR: 0.27; 95% CI: 0.12, 0.64) lower hazard of metastasis after tumor resection (+/−RT) (Supplementary Table S2). Therefore, we recommend neo/adjuvant chemotherapy for patients with tumor size 5 cm or greater because of the poor survival outcomes in this group of patients, as well as the significant benefits of chemotherapy shown with delayed progression since diagnosis and delayed metastasis after definitive treatment of the primary tumor. Considering the overall poor prognosis of patients with intrathoracic synovial sarcoma, one could consider chemotherapy for patients with tumors less than 5 cm on a case-by-case basis. Also, with the marginal significance of chemotherapy benefits among patients with <5 cm tumor size (HR:0.30; 95% CI: 0.08, 1.13) (Supplementary Table S2), future studies with larger sample size should evaluate the effect of chemotherapy in relation to tumor size less than 5 cm in detail.

Our study is subject to some limitations. One limitation inherent to retrospective reviews is that treatment was not randomized. We also did not have a sufficiently large cohort to evaluate what type of chemotherapy might be more effective. Therefore, future studies should be conducted with a larger sample size using multi-institutional data because of the rarity of this disease. Moreover, more data on the differentiation of fusion genes (SS18::SSX1 or SS18::SSX2) could be helpful in more precise risk stratification. Additionally, the resection margin variable had missing data, which could provide more insight into the definitive treatment of primary tumors. However, a major strength of this analysis is that the cohort is the largest of patients with intrathoracic SS to be published to date.

## 5. Conclusions

In conclusion, the outcome of patients with intrathoracic SS is significantly worse than SS in more common sites such as the extremities. Chemotherapy for localized tumors ≥ 5 cm is associated with better survival, irrespective of tumor origin within the chest. Considering the fact that intrathoracic SS patients die of metastatic disease, we recommend that patients with 5 cm or greater primary tumor size should receive perioperative systemic therapy to improve progression and metastasis-free survival.

## Figures and Tables

**Table 1:** Demographic and clinical characteristics of intrathoracic synovial sarcoma patients presenting with localized disease at diagnosis (n = 63).

Characteristics	Patients with Localized Disease at Diagnosis (n = 63)
Age at diagnosis (y) Median (range)	43 (18–77)
<45	36 (57)
≥45	27 (43)
Total	63
Sex	
Female	36 (57)
Male	27 (43)
Total	63
Race and ethnicity	
White	57 (90)
Others *	6 (10)
Total	63
Smoking history	
Never	41 (65)
Yes	19 (30)
Unknown	3 (5)
Total	63
Primary site	
Lung	43 (68)
Lung with involvement of pleura	2 (3)
Chest wall	3 (5)
Mediastinum	15 (24)
Total	63
Tumor size (cm) Median (range)	7 (1–18)
<5	15 (24)
5–10	25 (40)
≥10	15 (24)
Unknown	8 (13)
Total	63
Subtype	
Monophasic	47 (75)
Biphasic	6 (10)
Poorly differentiated	5 (8)
Unknown	5 (8)
Total	63
Fusion gene	
Present **	40 (63)
Not tested	23 (37)
Total	63
Stage	
Stage ll	17 (27)
Stage lllA	26 (41)
Stage lllB	12 (19)
Stage lV	0
Unknown	8 (13)
Total	63
Tumor resection	
Non-resected	1 (2)
Resected	62 (98)
Total	63
Final surgical resection margin	
Negative	35 (56)
Positive	13 (21)
Unknown	14 (23)
Total (Patients who had resection)	63
Neo/adjuvant radiation therapy	
No	44 (71)
Yes	18 (29)
Total (Patients who had resection)	63
Timing of radiation therapy	
Neoadjuvant	2 (11)
Adjuvant	15 (83)
Unknown	1 (6)
Total	18
Neo/adjuvant chemotherapy	
No	19 (31)
Yes	43 (69)
Total (Patients who had resection)	62
Timing of chemotherapy	
Neoadjuvant	17 (40)
Adjuvant	26 (60)
Total	43
Chemotherapy regimen	
Doxorubicin and ifosfamide	34 (79)
High-dose ifosfamide	1 (2)
Gemcitabine and docetaxel	1 (2)
Other	7 (16)
Total	43
Local recurrence	
No	48 (77)
Yes	14 (23)
Total (Patients who had resection)	62
Metastasis	
No	16 (25)
Yes	47 (75)
Total	63

Note: * Other races and ethnicities included Hispanic or Latino and Asian. ** SS18::SSX1—5 (13%), SS18::SSX2—7 (18%), Unknown—28 (70%).

**Table 2:** Comparison of median survival times for intrathoracic synovial sarcoma patients presenting with localized disease.

Variables (Log-Rank *p*)	Median PFS (Years)	Median OS (Years)	Median MFS (Years)
Entire Cohort (n = 63)			
Age at diagnosis (Years)	0.565	0.461	0.942
<45	0.92	3.00	1.00
≥45	1.25	3.58	1.33
Sex	0.292	0.880	0.992
Female	1.08	3.50	1.00
Male	1.17	3.00	1.33
Race and ethnicity	0.356	0.851	0.778
Other	0.92	2.42	1.08
White	1.17	3.50	1.08
Smoking history	0.768	0.294	0.538
Never	1.08	3.00	1.08
Yes	1.17	3.92	1.08
Tumor origin	0.191	0.716	0.490
Lung	1.08	3.33	1.08
Lung with pleura	0.83	2.25	0.67
Chest wall	1.33	3.58	1.08
Mediastinum	1.67	2.67	1.83
Tumor size (cm)	0.472	0.014 *	0.875
<5	1.25	6.33	1.33
≥5	1.17	2.50	1.17
Subtype	0.674	0.436	0.723
Monophasic	1.17	3.33	1.08
Biphasic	1.08	2.58	0.58
Poorly differentiated	2.92	2.67	0.75
Tumor resection	0.182	0.510	-
No	-	-	-
Yes	1.17	3.33	-
Patients with surgical resection performed (n = 62)			
Final resection margin	0.459	0.044 *	0.335
Negative	1.25	3.58	1.33
Positive	0.83	3.83	0.50
Unknown	1.08	2.25	1.00
Neo/adj radiation therapy	0.801	0.315	0.119
No	1.17	3.33	1.17
Yes	1.08	3.50	0.67
Neo/adj chemotherapy	0.0002 *	0.187	0.005 *
No	0.58	2.58	0.50
Yes	1.25	3.50	1.33

Note: * Log-rank *p* < 0.05.

**Table 3:** Cox proportional hazard regression results for the association of tumor origin with PFS, OS, and MFS among intrathoracic synovial sarcoma patients presenting with localized disease (n = 63).

Variables	PFS		OS		MFS	
	Univariate Analysis HR (95% CI)	Multivariable Analysis HR (95% CI)	Univariate Analysis HR (95% CI)	Multivariable Analysis HR (95% CI)	Univariate Analysis HR (95% CI)	Multivariable Analysis HR (95% CI)
Age at diagnosis (Years)						
<45	Reference	-	Reference	-	Reference	-
≥45	0.85 (0.49, 1.49)	-	1.24 (0.69, 2.26)	-	1.02 (0.55, 1.89)	-
Sex						
Female	Reference	-	Reference	-	Reference	-
Male	1.34 (0.76, 2.35)	-	0.96 (0.52, 1.75)	-	1.00 (0.54, 1.87)	-
Race and ethnicity						
Other	Reference	-	Reference	-	Reference	-
White	0.68 (0.29, 1.60)	-	0.91 (0.32, 2.56)	-	0.88 (0.34, 2.25)	-
Smoking history						
Never	Reference	-	Reference	-	Reference	-
Yes	1.09 (0.60, 1.98)	-	0.71 (0.37, 1.37)	-	1.23 (0.63, 2.40)	-
Tumor origin						
Lung	Reference	Reference	Reference	Reference	Reference	Reference
Lung with pleura	2.00 (0.47, 8.54)	2.94 (0.66, 13.00)	2.28 (0.53, 9.89)	2.11 (0.27, 16.21)	2.15 (0.50, 9.31)	3.27 (0.72, 14.85)
Chest wall	0.88 (0.27, 2.91)	1.27 (0.37, 4.35)	1.11 (0.33, 3.70)	1.02 (0.31, 3.42)	1.59 (0.48, 5.29)	2.37 (0.67, 8.37)
Mediastinum	0.54 (0.27, 1.07)	0.80 (0.38, 1.66)	1.11 (0.54, 2.29)	0.86 (0.38, 2.04)	0.77 (0.35, 1.70)	1.11 (0.47, 2.61)
Tumor size (cm)						
<5	Reference	-	Reference	Reference	Reference	-
≥5	1.28 (0.64, 2.53)	-	2.68 (1.17, 6.15)	2.66 (1.16, 6.11)	1.06 (0.51, 2.22)	-
Subtype						
Monophasic	Reference	-	Reference	-	Reference	-
Biphasic	1.13 (0.44, 2.89)	-	1.60 (0.62, 4.12)	-	1.46 (0.56, 3.77)	-
Poorly differentiated	0.62 (0.19, 2.03)	-	1.64 (0.57, 4.70)	-	1.07 (0.25, 4.48)	-
Tumor resection						
No	Reference	-	Reference	-	Reference	-
Yes	-	-	0.52 (0.07, 3.87)	-	-	-
Final resection margin						
Negative	Reference	-	Reference	-	Reference	-
Positive	1.48 (0.75, 2.94)	-	1.38 (0.65, 2.96)	-	1.72 (0.77, 3.75)	-
Unknown	1.29 (0.65, 2.57)	-	2.43 (1.17, 5.02)	-	1.38 (0.64, 2.96)	-
Neo/adj radiation therapy						
No	Reference	-	Reference	-	Reference	-
Yes	0.93 (0.52, 1.67)	-	1.38 (0.73, 2.60)	-	1.66 (0.86, 3.20)	-
Neo/adj chemotherapy						
No	Reference	Reference	Reference	-	Reference	Reference
Yes	0.34 (0.18, 0.62)	0.33 (0.17, 0.65)	0.67 (0.36, 1.24)	-	0.41 (0.21, 0.79)	0.35 (0.17, 0.73)

PFS: Progression-free survival (date of diagnosis to date of recurrence/metastasis); OS: Overall survival (date of diagnosis to date of death); MFS: Metastasis-free survival (date of definitive treatment to date of metastasis); HR: Hazard Ratio; CI: Confidence Interval.

## Data Availability

The data presented in this study are available on request from the corresponding author due to privacy, legal, and ethical reasons.

## Supplementary Materials

The following supporting information can be downloaded at https://www.mdpi.com/article/10.3390/cancers17050745/s1, Supplementary Figure S1. Overall survival by primary tumor size (5–10 vs. ≥10 cm) for intrathoracic SS patients presenting with localized disease. Supplementary Table S1. Relationship between tumor size and perioperative radiation therapy in patients who had surgical resection (n = 64). Supplementary Table S2. Cox proportional hazard regression for neo/adjuvant chemotherapy by primary tumor size.
