# Supplementary material for: Primary Intrathoracic Synovial Sarcoma: An Analysis of Outcomes of This Rare Disease
Source: Cancers (Basel). 2025 Feb 22;17(5):745. doi: 10.3390/cancers17050745 (PMC11898520; doi:10.3390/cancers17050745)
Supplement: Supplementary file 1 [file cancers-17-00745-s001.zip › cancers-3431206-supplementary.pdf]

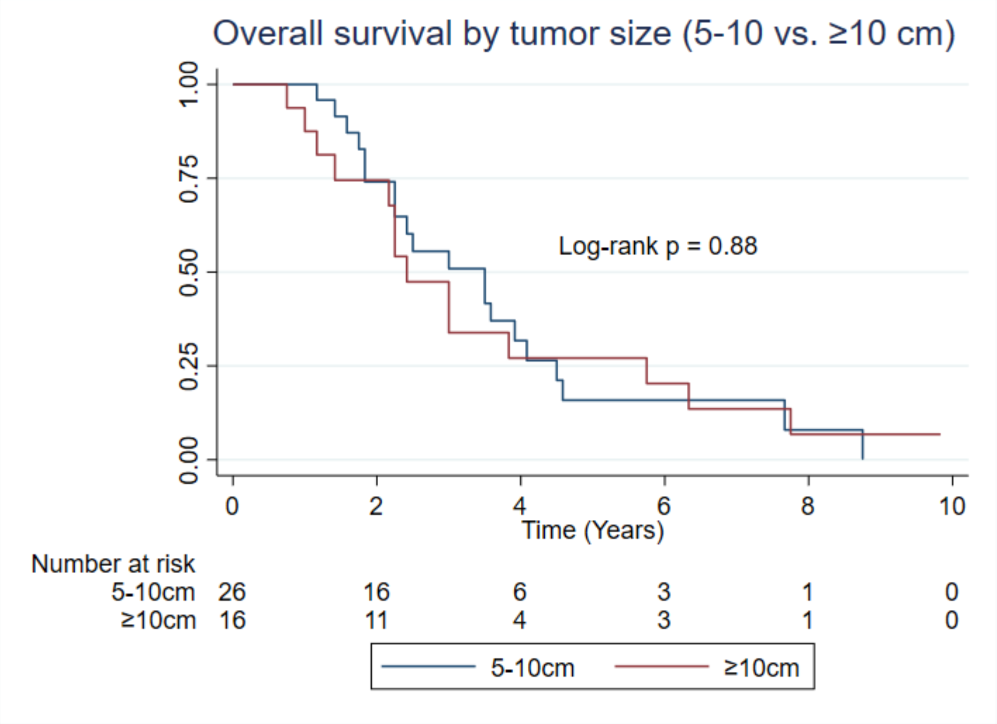

Supplementary Figure S1. Overall survival by primary tumor size (5-10 vs.≥10 cm) for intrathoracic SS patients presenting with localized disease.

Supplementary Table S1. Relationship between tumor size and perioperative radiation therapy in patients who had surgical resection (n = 64).

Tumor Size	Neo/adjuvant Radiotherapy		P-value
	No (n = 45)	Yes (n = 19)	
<5 cm	13 (29)	1 (5)	0.039
5-10 cm	19 (42)	7 (37)	
≥10 cm	10 (22)	6 (32)	
Unknown	3 (7)	5 (26)	

Supplementary Table S2. Cox proportional hazard regression for neo/adjuvant chemotherapy by primary tumor size.

	PFS		MFS	
	Tumor size		Tumor size	
	<5 cm HR (95% CI)	≥5 cm HR (95% CI)	<5 cm HR (95% CI)	≥5 cm HR (95% CI)
Neo/adjuvant chemotherapy				
No	**Reference**	**Reference**	**Reference**	**Reference**
Yes	**0.30 (0.08, 1.13)**	**0.47 (0.23, 0.98)**	**0.87 (0.23, 3.28)**	**0.37 (0.17, 0.82)**

PFS: Progression-free survival (Date of diagnosis to date of recurrence/metastasis)
MFS: Metastasis-free survival (Date of definitive treatment to date of metastasis)
HR: Hazard Ratio
CI: Confidence Interval
